# Prevalence and predictors of wind energy opposition in North America

**DOI:** 10.1073/pnas.2302313120

**Published:** 2023-09-25

**Authors:** Leah C. Stokes, Emma Franzblau, Jessica R. Lovering, Chris Miljanich

**Affiliations:** ^a^Department of Political Science, University of California, Santa Barbara, CA 93106-9420; ^b^Fastest Path to Zero Initiative, University of Michigan, Ann Arbor, MI 48109; ^c^Public Sector Consulting, Gallup, Washington, DC 20004

**Keywords:** climate change, renewable energy, energy opposition, wind energy

## Abstract

Addressing climate change requires societies to transition away from fossil fuels toward low-carbon energy, including renewables. Unfortunately, large wind projects have proven politically controversial, with groups opposing them across advanced economies. To date, there are few large-scale, systematic studies to identify the prevalence and predictors of opposition to wind energy projects. Here, we analyzed a dataset of wind energy projects across the United States and Canada between 2000 and 2016. We found that during this period, in the United States, 17% of wind projects faced significant opposition, and in Canada, 18% faced opposition, with rates in both countries growing over time. Opposition was concentrated regionally in the Northeastern United States and in Ontario, Canada. In both countries, larger projects with more turbines were more likely to be opposed. In the United States, opposition was more likely and more intense in areas with a higher proportion of White people, and a smaller proportion of Hispanic people. In Canada, opposition was more likely and more intense in wealthier communities. The most common tactics used to oppose wind energy were court cases, legislation, and physical protests. The number of people engaging in opposition to wind projects is likely small: Across articles that cited the number of individuals engaging in protests, the median number was 23 in the United States and 34 in Canada. When wealthier, Whiter communities oppose wind projects, this slows down the transition away from fossil fuel projects in poorer communities and communities of color, an environmental injustice we call “energy privilege.”

## Significance

Local opposition poses a significant barrier to rapid deployment of wind energy. Our study looks comprehensively at opposition to wind projects across the United States and Canada between 2000 to 2016. Larger projects were more likely to be opposed. In the United States, opposition was more likely and more intense in areas with a higher proportion of White people, and a lower proportion of Hispanic people; in Canada, the same pattern held for wealthier communities. The names in articles associated with US opposition were overwhelmingly likely to be White. This suggests an environmental justice challenge we term “energy privilege,” wherein the delay and cancellation of clean energy in wealthier, Whiter communities leads to continued pollution in poorer communities, and communities of color.

Across North America, the electricity system is undergoing a shift away from conventional polluting technologies, like coal and fossil gas power plants, toward renewable energy resources (1). Wind energy is by far the most common renewable energy technology. In the United States and Canada, wind energy grew rapidly from less than 1% of electricity generation in 2000 in both countries, to 8% and 6% in 2020, in the United States and Canada, respectively (2). This shift has public health and climate benefits, reducing local air pollution and carbon pollution. Unfortunately, wind energy has proven politically controversial at the local scale. Across North America (3–5) and Europe (6–8) wind projects have faced local opposition. Resistance to wind energy development presents a significant challenge for the energy transition, as the rate of wind energy deployment needs to accelerate rapidly to meet decarbonization targets.

The prevalence of political opposition to proposed wind projects is not well understood. While some research has examined specific cases of wind opposition (9, 10), most work has focused on relatively small geographic areas (11–14). Existing research typically relies on case studies, often using surveys to assess individual-level perceptions of wind energy at the local level. In a few cases, research has examined larger samples (15). For example, Giordono et al. (16) researched 53 proposed wind farm developments in the Western United States to understand factors driving opposition. They found 36% of the proposed wind projects they studied faced public opposition in some form, with 8% experiencing a protest. Such a high prevalence of wind opposition contrasts with polling that typically finds public support for expanding wind energy is over 80% (17). Hence, there is a mismatch between broad public support for wind energy and local opposition to specific projects (5). In this research, we find that opposition was present in 17% of wind energy projects in the United States, and 18% in Canada, between 2000 and 2016.

Existing research proposes a wide variety of factors that could predict opposition to wind energy projects, including demographics; project characteristics; location; perceived economic and environmental costs and benefits; and, process (18). Demographics may play an important role in local acceptance. One study that relied on a national survey found higher support for wind energy among males and White people; and lower support from those who identified as Republicans, were more conservative, or lived in the Northeast (19). Several other papers find a political divide, with greater opposition from conservatives (20) and greater support from liberals (21). However, other studies find only borderline evidence of party identification effects (22). In Canada, studies have found that partisanship plays a role in support for wind energy (5, 23).

Project characteristics, such as the overall project size, and the size or height of the turbines, could also affect support. Existing research has found that noise and disruptions to the viewscape—which are correlated with project size—predict opposition (3, 20). Numerous studies find aesthetics are key to wind project support (24–26). For example, Fergen and Jacquet (24) find that local residents perceive turbines to be more beautiful in motion than static—hence in places with higher wind speed, we may expect to find greater support for wind projects. Notably, such projects would also generate greater economic activity.

Wind projects’ location may also affect support, with some regions being more supportive or hostile to projects. Several studies have found that individuals living closer to wind energy facilities are less supportive of projects (5, 19, 27), suggesting wind projects in remote areas may receive higher support. Land use may also affect support. For example, Bessette and Mills (28) looked at 69 wind projects in the Midwestern United States and found lower opposition in areas that had more production-oriented farming and fewer natural amenities. Research on setbacks—how far wind turbines must be located away from homes, roads, or property lines—has found mixed results (20). Overly large setbacks can essentially prohibit wind projects in a region (1), whereas prohibitions on local setback ordinances may facilitate more wind development but lead to larger public opposition (29).

Wind projects’ perceived economic costs and benefits may also predict opposition (14, 30). If wind projects are seen as providing local benefits, such as jobs or reduced energy costs, we should expect higher support; if they are perceived as imposing costs, such as lowering home prices, we should expect lower support. Economic hardship, measured as high unemployment, appears to mitigate opposition to energy infrastructure perhaps because of an interest in job creation (31). Experimental evidence from surveys suggests that providing environmental or economic information can influence support for wind power, but such priming can also strengthen opposition for those who already believe wind projects have negative environmental or economic impacts (19, 22). Perceived impact on wildlife habitat is also shown to predict opposition (32).

Finally, the process to develop wind projects can also shape public acceptance. Stakeholder engagement is important to minimizing opposition (33–35). A recent study by Susskind et al. (36) argues that stakeholder perspectives should be incorporated in the siting process as early as possible. Additional research finds that lack of perceived fairness and equity, particularly in the decision-making processes at the local level, increases opposition (15, 37–39). For example, Mills et al. (40) found that residents perceived greater benefits from wind projects if they think the planning process was fair.

To understand the prevalence and predictors of wind opposition in North America, we created a comprehensive dataset of wind projects across the United States and Canada from 2000 to 2016. We included both successful and unsuccessful opposition in our analysis, as even delays can slow down wind energy deployment, increasing costs and undermining progress on cutting pollution. We collected information on demographics and project characteristics. We then compiled almost 36,000 news articles that mentioned specific wind energy projects in either country. We used human coders to identify whether wind energy projects faced opposition and what form the opposition took. News articles were chosen as a means to identify opposition to wind projects as they are likely the only comprehensive tool available to do so. We defined opposition as physical protests, legal actions, legislation, and/or letters to the editor, all aimed at preventing the project’s completion. Comparing the differences in means across projects with and without opposition and using multivariate ordinary least squares (OLS) regression, we identified predictors associated with opposition to wind energy projects throughout the United States and Canada.

Across both countries, opposition to wind energy has grown over time. It is also concentrated regionally: in the Northeastern United States, and in Ontario, Canada. The most common tactics used to oppose wind projects were legal challenges, legislation, and in Canada protests. In both countries, opposition was more likely for larger projects with more turbines. In Canada, opposition was more likely and more intense in wealthier areas. In the United States, opposition was less likely if wind projects were community owned. It was also more likely and more intense in Whiter areas, with smaller proportions of Hispanic people. Analyzing the names of people listed in articles describing opposition to US wind projects, we found that opponents were overwhelmingly likely to be White. In addition, the number of people involved in opposing wind projects was likely small: The median number of protesters mentioned in news articles was 23 in the United States and 34 in Canada. These findings have significant environmental justice implications. If wealthier, Whiter communities block wind projects, this slows the pace of the clean energy transition, lengthening the lifespan of polluting infrastructure in lower-income communities, and communities of color. Blocking clean infrastructure in wealthier, Whiter communities is a form of energy privilege that imposes costs on lower-income communities, and communities of color.

## Results

### Prevalence of Opposition toward Wind Energy Projects.

**Fig. 1.**
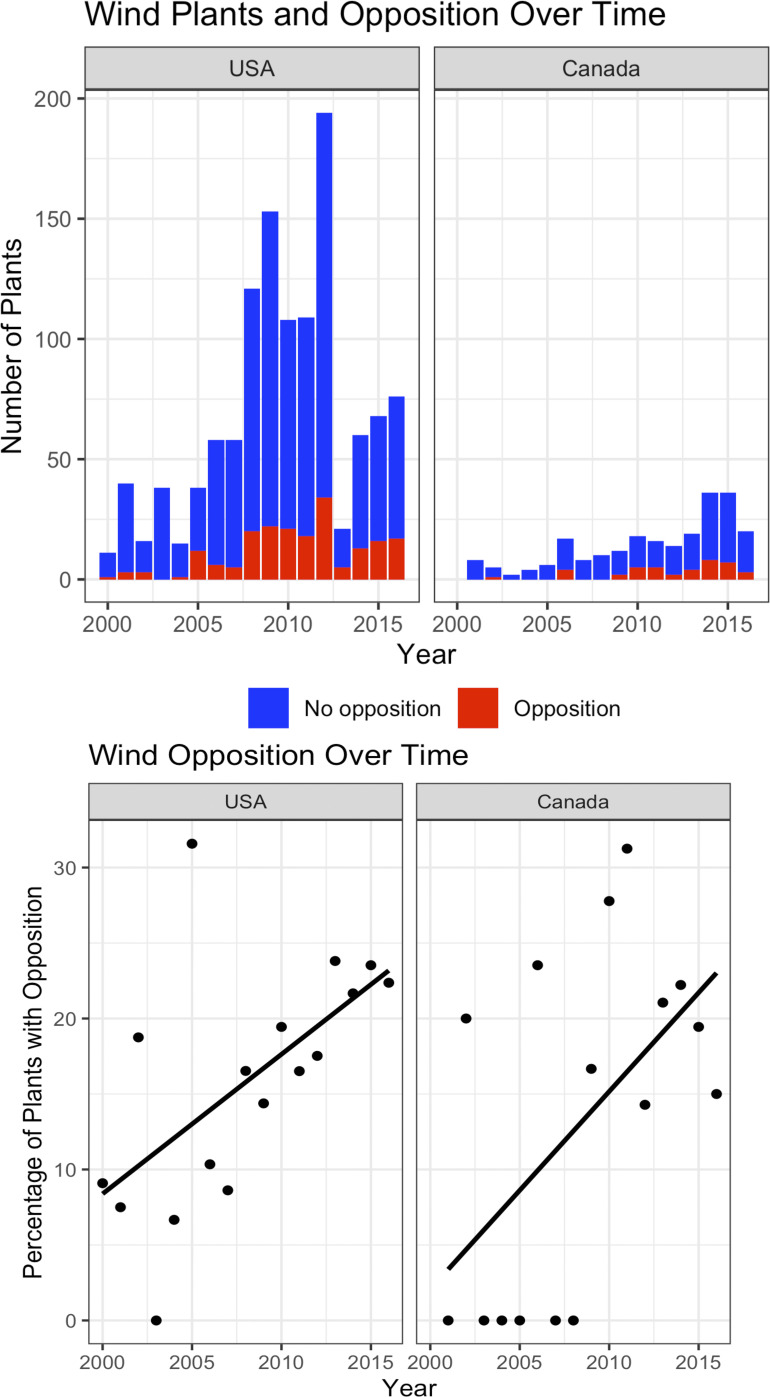
Top row: Number of wind plants in Canada and the United States over time, by year of operation. Projects that did not experience opposition are shown in blue, those that did are shown in red. Bottom row: Percentage of plants that experienced opposition in both countries over time, including a linear trend line.

Between 2000 and 2016, we found that 197 out of 1,184 total projects in our US dataset (17%) experienced opposition. Over this same time period, 41 out of 231 total projects in our Canadian dataset (18%) experienced opposition. Fig. 1 shows the number of new operational projects by year in both countries, along with the breakdown of how many experienced opposition in each year. As wind energy has grown, so too has opposition to projects.

**Fig. 2.**
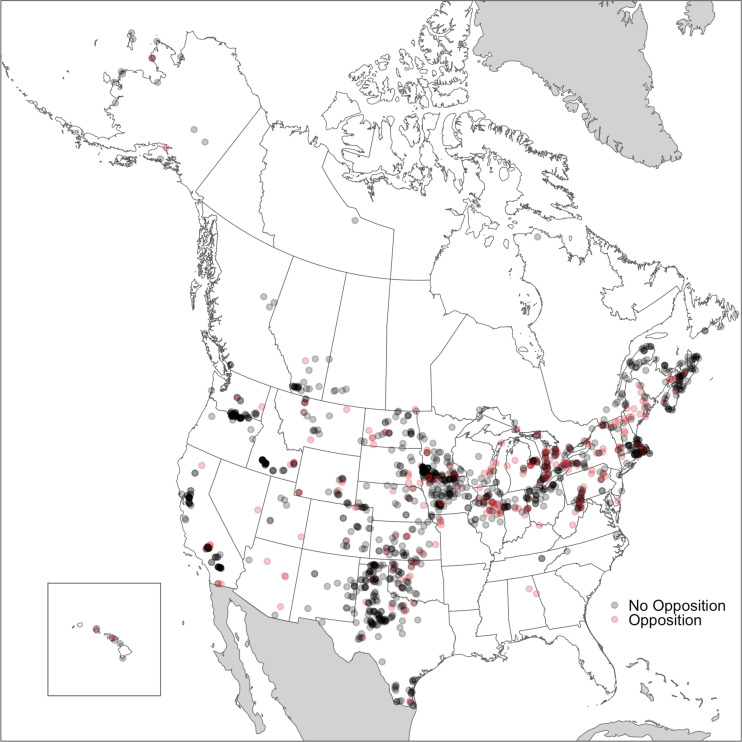
Spatial distribution of wind energy projects and opposition in the United States of America and Canada. Projects that experienced opposition are shown in red. Darker shades indicate a larger concentration of plants in that specific area.

As Fig. 2 shows, in the United States during this period, wind projects were heavily concentrated in the middle of the country, in the area stretching from Minnesota to Texas (Fig. 2). They were also interspersed throughout the West, Mountain West, and Northeast. There were few projects in the Southeast, in part because those states have never passed Renewable Portfolio Standard laws, and have comparatively low wind speeds. In Canada, wind projects were concentrated in Ontario due to the province’s supportive policies for renewable energy in the 2000s (41). We found that opposition was concentrated in the Northeastern United States and in Ontario, Canada. While only 14% of all US wind projects in this period were located in the Northeast, 25% of projects facing opposition were in the region. Overall, 31% of the projects located in the Northeastern United States experienced opposition. Similarly, while 38% of Canada’s wind projects were located in Ontario, 78% of projects experiencing opposition were in the province. Overall, 37% of projects located in Ontario experienced opposition.

Our data on wind farms in the United States were biased toward projects close to completion. As a robustness check, we also pulled data on opposition to wind farms from a study published by the Sabin Center for Climate Change Law at Columbia University (42). From this report, we found 42 wind projects that faced some form of opposition between 2000 and 2016. Of these 42 projects, 25 were not already in our dataset. The likely explanation for their initial exclusion was that most were canceled or stalled and have yet to start construction (one was an offshore wind project).

### Predictors of Opposition toward Wind Energy Projects.

Our analysis aimed to understand what variables predicted opposition to wind projects. Table 1 provides demographic and project characteristics for each census tract that a wind project was located in, separated by those with and without opposition. In both countries, larger projects, with more turbines were more likely to face opposition. In the United States, taller turbines were also associated with opposition. While turbine height data were not easily available in Canada, we assume the relationship holds there since turbine height is a function of project size and number of turbines, both of which significantly predict opposition in Canada.

**Table 1.** Demographic and project characteristics for wind projects with and without opposition in the United States of America and Canada

	No Opposition	Opposition	*t*-statistic
	The United States		
Population Density (per $mi^{2}$)	194	121	−1.69${}^{*}$
Household Median Income	$49,500	$50,300	0.78
Percent White	81%	89%	7.12${}^{***}$
Percent Hispanic	13%	6%	−7.74${}^{***}$
Percent Black	2%	2%	−1.38
Percent Community Ownership	28%	19%	−2.80${}^{***}$
Percent GOP Votes	67%	68%	0.71
Capacity (MW)	70	110	4.36${}^{***}$
Number of Turbines	39	60	4.16${}^{***}$
Turbine Height (in ft)	221	239	3.21${}^{***}$
N	987	197	
	Canada		
Population Density (per $mi^{2}$)	21	8	−1.46
Household Median Income	$67,000	$73,000	2.59${}^{**}$
Percent White	97%	98%	0.94
Percent Liberal	42%	35%	−2.49${}^{**}$
Capacity (MW)	44	80	2.78${}^{***}$
Number of Turbines	23	38	2.35${}^{**}$
N	190	41	

Note: Column 4 provides *t*-statistics for difference-in-means tests. Due to rounding, percents may not sum to 100.

${}^{*}$P<0.1; ${}^{**}$P<0.05; ${}^{***}$P<0.01.

In the United States, wind projects experiencing opposition were located in areas with larger percentages of White people, and lower percentages of Hispanic people. These race and ethnicity variables are by far the strongest predictors of opposition. In addition, US projects with some form of community ownership were less likely to face opposition. In general, community-owned wind projects involve significant profit sharing with local entities, that goes beyond standard lease payments to landowners. Notably, opposition was not associated with partisanship in the United States.

In Canada, we found that wind projects were more likely to experience opposition in areas with higher median incomes. Unlike the United States, race was not a significant predictor in Canada, likely because places where wind projects were developed were on average 97% White, leaving little room for variation. Instead, partisanship was significant in Canada: Places experiencing opposition had lower support for the Liberal Party. This is not surprising since the Liberal party developed the key policy supporting wind energy in Ontario that led to rapid growth in wind energy but which also removed local opponents’ ability to block projects, leading to concentrated albeit small-scale backlash (4, 5).

Since our data focus on projects close to completion, as a robustness check, we included in our US dataset wind projects that were canceled, drawing on a study published by the Sabin Center for Climate Change Law at Columbia University (42). From this report, we found an additional 25 wind projects that were opposed, all but one of which were canceled. As shown in *SI Appendix*, Table S3, we find that race and ethnicity predicted project cancellation–canceled projects were in communities that were on average 95% white. These canceled projects were also larger than other opposed projects, at nearly 200 MW on average.

For the US data, we also used a name classifier to examine the likely race of individuals listed in news articles related to opposition to wind projects. Of the 2,157 names we identified in the data, we found that there was a 92.4% chance that an individual listed was White. This probability is higher than the White population in census tracts with wind projects (89%). No other racial group had a greater than 4% chance of an opponent being from that group. This suggests that wind energy opponents are predominantly White.

We also used OLS regression models to examine predictors of opposition to wind projects in both the United States and Canada (*SI Appendix*, Tables S1 and S2). Overall, the findings are convergent. As is also shown in Fig. 2, the OLS results suggest that opposition was highly concentrated regionally. Wind projects in the Northeastern United States experienced opposition at around 19 percentage points (p.p.) higher rates as compared to the Midwest. Similarly, wind projects located in Ontario experienced opposition at around 26 p.p. higher—and those in British Columbia around 28 p.p. lower—as compared to Atlantic Canada. For the United States, areas with larger proportions of Hispanic people were less likely to oppose wind projects. Substantively, a project located in an area where the Hispanic population was 10 p.p. larger was 2 to 3 p.p. less likely to experience opposition, depending on the specific model. Substantively, this effect is large: Given the baseline rate of opposition in the United States, such a shift would decrease the likelihood of opposition to a wind project by almost 20%. In the United States, taller turbines were also more likely to be opposed, although substantively the effect is small—a turbine that was 10 feet taller would be 0.3 p.p. more likely to experience opposition. For Canada, median income was significant, with wealthier areas more likely to oppose wind projects. Substantively, a project located in an area where the median income was $10,000 greater had a 4 to 5 p.p. increase in experiencing opposition. Again, this effect is substantive, representing an almost 30% increase in the probability of a project being opposed, given the baseline rate of opposition in Canada.

### Tactics and Intensity of Opposition to Wind Projects.

We also aimed to understand the tactics used to oppose wind projects across North America. We coded newspaper articles for whether they mentioned the use of the courts, legislation, physical protests, or letters to the editor to oppose projects. We defined using the courts as a legal challenge to a wind project, and legislation as the attempt to enact laws, policies, or codes to prevent the wind project from being built. We only recorded use of a physical protest where the number of protesters was mentioned in an article. Table 2 shows the relative frequency of each tactic. In the United States, courts were the dominant mode of opposition, followed by legislation, then physical protest, then letters to the editor. In Canada, physical protest was the dominant mode of opposition followed by courts, legislation, and letters to the editor. We also examined how many people were engaging in protests when mentioned. Across articles that estimated the number of individuals engaging in protests, the median number was 23 in the United States and 34 in Canada. While the accuracy of newspaper protest estimates is open to debate (*SI Appendix*), the order of magnitude is likely correct and suggests that only a small number of people were mobilizing to block clean energy projects in any given location.

**Table 2.** Opposition tactics in the United States of America and Canada

Tactic	US	Canada
Courts	68%	61%
Legislation	56%	39%
Physical Protest	37%	76%
Letters to the Editor	20%	2%
Scale of Protests	US	Canada
Mean Participants	99	29
Median Participants	23	34

Shown are the percentage of projects that experienced each type of opposition tactic. Percentages were calculated across projects that *experienced* opposition. Mean and median number of individuals who opposed were calculated only for projects that experienced opposition *and* for whom the estimated number of individuals opposing the project was mentioned.

To understand what variables were associated with the intensity of opposition to wind projects, we constructed an “opposition score.” This is an intensity measure, from 0 to 4, for how many opposition tactics were used to resist a specific project. For example, a project with an opposition score of 4 involved a court case, legislation, physical protest, and letters to the editor; a project with an opposition score of 0 involved none of these specific tactics. Table 3 shows the relationship between demographics and project characteristics and the opposition score. In the United States, Whiter census tracts opposed wind projects more intensely, with a positive correlation of 0.18. Wind projects located in areas with a greater share of Hispanics opposed projects less intensely, with a negative correlation of 0.12. Hence, not only did wind projects in Hispanic areas experience less opposition, but they also faced a narrower set of opposition tactics. Just as GOP vote share did not predict opposition, it also did not predict the intensity of opposition. Taller turbines were also more likely to face more intense opposition in the United States, with a positive correlation of 0.44. In Canada, wealthier communities had a higher opposition score, with a positive correlation of 0.23. Areas with a lower share of Liberal party support used more tactics to oppose projects. In Canada, larger projects with more turbines were also correlated with more intense opposition.

**Table 3.** Opposition score in the United States of America and Canada

Opposition Score	0	1	2	3	4	r
	The United States					
Population Density (per $mi^{2}$)	330	120	34	110	110	-0.10
Household Median Income	48,900	50,800	48,800	52,700	50,100	0.04
Percent White	86%	89%	87%	94%	94%	0.18
Percent Hispanic	7%	6%	8%	2%	3%	-0.12
Percent Black	3%	1%	2%	1%	1%	-0.06
Percent GOP Votes	68%	68%	69%	69%	64%	-0.03
Percent Community Ownership	26%	25%	5%	23%	14%	-0.10
Capacity (MW)	102	91	149	113	83	0.02
Number of Turbines	63	50	79	61	45	-0.01
Turbine Height (in ft)	196	204	268	275	268	0.44
N	26	65	51	33	22	
	Canada					
Population Density (per $mi^{2}$)	4	9	9	7		0.04
Median Income (in CAD)	60,900	73,700	72,600	77,000		0.23
Percent White	98%	98%	98%	97%		-0.03
Percent Liberal Votes	52%	38%	29%	36%		-0.23
Capacity (MW)	45	44	93	111		0.32
Number of Turbines	21	22	43	53		0.32
N	3	11	19	8	0	

The variable indicates how many of the four opposition tactics a wind project experienced: court cases, legislation, physical protest, and letters to the editor. Means are reported for demographics and project characteristics. The column titled “r” reports the correlation coefficients between each variable and the opposition score.

## Discussion

Overall, we found widespread opposition to wind energy projects across North America that was growing over time. In the first half of our dataset, between 2000 and 2008, 13% and 8% of wind projects in the United States and Canada experienced opposition, respectively. Between 2009 and 2016, those numbers grew to 19% and 21%—in other words, one in five projects was opposed. Were this study replicated with data from 2016 onward, we would expect the level of opposition to wind projects to be higher.

Across both countries, we found that opposition was concentrated regionally. Wind projects located in the Northeastern United States and in Ontario, Canada were much more likely to experience opposition than projects located in other areas. Larger projects, with more turbines—and in the United States those with taller turbines—were more likely to be opposed. Data on turbine height were not available in Canada, though we expect these results to hold as turbine height is a function of project size and number of turbines. In Canada, wealthier areas, and places with fewer Liberal Party supporters were more likely to oppose projects. In the United States, there was no association between partisanship and opposition to wind projects.

In the United States, race and ethnicity appear to play a significant role in predicting whether opposition occurs and the intensity of that opposition. Wind projects in areas with a higher percentage of White people and a lower percentage of Hispanic people were more likely to face resistance, and that opposition was more intense, with a larger number of tactics. In addition, the names in newspaper articles associated with wind opposition were overwhelmingly likely to be White. This may reflect racial differences in political participation and resources. Whites are typically more politically involved than other racial groups (43, 44).

Across both countries, we found that small numbers of people turned out to protest wind projects—typically around 20 to 30 people—suggesting that small numbers of White and wealthier people in rural areas are blocking wind projects. Notably more recent investigations have suggested that in the United States, many of the small groups responsible for opposition to wind projects are shown to indirectly receive funding from fossil fuel companies through far-right think tanks—a dynamic that likely grew in the period after 2016 (45, 46). This is a key piece of context that is largely missing from articles covering opposition to specific projects. Further research should examine the relationship between fossil fuel interests and wind energy opposition.

While previous research has convincingly shown that the siting process is important to acceptance (36, 40), this is largely outside the scope of our analysis. It is likely that if there was a measure of the quality of the process, this would predict opposition. The closest proxy that we have for process is a measure of community ownership for American wind projects. In line with previous research, we found that community ownership was associated with less opposition. This suggests that sharing profits from wind projects with the community will decrease opposition. That said, this measure of community ownership is too crude to unpack differences in process in detail. Future research that examines differences in siting processes across a large number of wind projects would be valuable.

Ultimately, our results speak to environmental justice. Classic and contemporary research has shown that polluting facilities are placed in communities of color, and low-income communities (47–49). As Tessum et al. (50) show, in the United States, White communities consume polluting goods and services, but the harm from this consumption overwhelmingly falls on Black and Hispanic groups. Building on this work, we find that Whiter and wealthier communities are slowing down and blocking wind projects across North America. Opposition to clean energy is a privilege. It imposes pollution burdens on poorer communities and communities of color, as it slows down the transition away from fossil fuel electricity sources overwhelmingly placed in their backyards (51). The impacts of this delay can be felt for generations. For example, Colmer and Voorheis (52) found that children whose parents experienced lower air pollution when the Clean Air Act was implemented were healthier, richer, and more likely to attend college. Similarly, Manduca and Sampson (53) found that children exposed to higher levels of air pollution experienced lower adult incomes, and higher likelihoods of incarceration and teen pregnancy. Overall, these small groups of wealthier and Whiter wind energy opponents in North America are slowing down the transition to clean energy by opposing wind projects in their backyards. This opposition represents a form of energy privilege that has dramatic air pollution impacts on low-income communities and communities of color. Given that polluting infrastructure also contributes to carbon and other greenhouse gas pollution, wind energy opposition also has effects globally, and on future generations.

## Materials and Methods

### Creating a Sample of Wind Projects.

For the United States, we identified the population of wind projects from the United States Energy Information Administration’s (EIA) 860 form data as well using data from the American Wind Energy Association (AWEA). From this list, we retained all wind projects that became operational between 2000 and 2016, as well as those that were in progress or canceled over this time period. However, we should note that data from EIA skews toward projects close to completion, as developers tend to submit Form 860 once they have a signed contract. To address this concern, we added additional wind projects from a study published by the Sabin Center for Climate Change Law at Columbia University (42). To ensure that the final list did not contain duplicates, projects were matched on latitude-longitude coordinates. We checked for duplicates using geographic location. In total, the final sample consists of 1,184 wind projects in the United States.

Project-level covariates were constructed from a variety of sources. These include project-level data from the EIA 860 form: project capacity size (MW); the number of turbines associated with the project; and turbine height. Where missing or inaccurate, project-level data were added using publicly available information. We used the coordinate locations of each project to identify its census tract. We then merged in tract-level 2010 census data including population density (per $mi^{2}$), median household income, White (non-Hispanic) share of population, Hispanic share of population, and Black (non-Hispanic) share of population. Finally, we merged in precinct-level 2016 presidential election returns from the Voting and Election Science Team at the University of Florida and Wichita State University (54). Again, we used coordinate locations to match each project to its precinct. We also purchased data on community ownership from the AWEA for the US wind projects and created a dummy variable indicating whether a wind plant was community owned.

For Canada, a complete database of operating wind projects was available from the Government of Canada. We merged this dataset with a list of proposed wind projects from the University of Alberta’s Canadian Renewable Energy Project Map. We narrowed the list of projects to those that began operations, or were still in progress, between 2000 and 2016 to match the US data, although we did not find any projects in 2000. The final dataset included 231 wind projects. Our data included attributes of wind projects such as location, total capacity in MW, and number of turbines. Demographic data were available at the census-tract level from Statistics Canada for the 2016 Census. Federal election data were available for the 2016 general election at the precinct level from Elections Canada.

### Measuring Opposition to Wind Projects.

To identify which wind projects faced opposition, we web-scraped two newspaper databases: LexisNexus and Wind Action. This approach resulted in 35,941 unique articles. To associate each newspaper article with a wind project, we used a fuzzy matching algorithm to match character strings in each newspaper article to wind projects that were operational between 2000 and 2016. Since a single project may have multiple colloquial names associated with it, fuzzy matching has the advantage of searching for variants of the original wind plant names. If a single wind project was mentioned in an article, it was associated in our database with that project. If multiple wind projects were mentioned, the article was associated with each wind project in the article.

After articles were matched to specific projects by name, they were assigned to a team of trained undergraduate student coders. To ensure consistency, the data were double-coded. Any articles that were not initially coded the same were adjudicated by a third coder who decided whether the article mentioned some form of opposition. Articles were grouped by the wind plant they pertained to, and aggregate statistics were calculated. Plants coded as facing opposition were checked once again to ensure that false positives did not exist in the data. In all, 4,283 US articles were found and coded that specifically mentioned anti-wind behavior associated with a specific project. Canadian articles were hand-coded by a team of undergraduate coders using the same process as the US data, with a total of 3,379 Canadian newspaper articles webscraped and coded for anti-wind behavior associated with a specific project.

Each coder was responsible for identifying whether the article mentioned a specific anti-wind action of some kind. We defined anti-wind actions as follows: physical opposition involving at least one person (e.g., protests, marches, picketing, mass presence at governmental meetings) and noted the number of protesters if specified; the use or attempted use of legislation or permitting to block projects; legal challenges and the use of courts to block projects; and letters to the editor that was explicitly opposed a project. If the coder identified examples of anti-wind behaviors, they coded the article accordingly. Articles could only be coded as anti-wind if they mentioned a specific wind project *and* mentioned opposition behaviors. We also created an opposition score, by using dummy variables for each of the four tactics mentioned above and summing them for each plant that experienced anti-wind activity.

We opted to only code explicit mentions of opposition as anti-wind activities in an effort to reduce the number of false positives. In our view, it was safer to be conservative when deciding whether an article mentioned anti-wind activity. Articles referencing noise complaints or animal and ecosystem studies did not count as being anti-wind. These were commonly referenced by anti-wind groups as evidence for why wind installations should not be built, but are not anti-wind actions in-and-of themselves. Because of this, we elected not to code articles referencing these issues as anti-wind unless they mentioned explicit anti-wind actions against a specific project. In addition, wind projects were only judged as being opposed if we could find a minimum of three newspaper articles describing opposition to a given project. To guard against false negatives, we aimed to gather as many newspaper articles as we could, ultimately collecting almost 36,000.

### Estimating Predictors with Differences in Means and OLS Regression Models.

Once we had every wind project categorized as experiencing opposition or not, we could look for relationships, using t tests for differences in means, and using OLS regressions. The OLS estimates show the change in the probability that opposition occurs given a specific covariate. Using the US data, we first modeled the relationship between the demographic characteristics of wind projects and opposition. These included tract-level Census covariates for population density (per 1,000 people per $mi^{2}$), household income (in $10,000), percent White (non-Hispanic), percent Black (non-Hispanic), and percent Hispanic as well as a measure of community ownership. The second OLS model adds project covariates including measures for the capacity size of the project in megawatts (MW), and the total number of turbines. A third model controls for percent of GOP vote in the 2016 presidential election, as well as region. In each model, we cluster standard errors at the census tract level. We used a similar approach with the Canadian data (see *SI Appendix* for full OLS results).

### Identifying Wind Project Opponents’ Names and Probable Race or Ethnicity.

To further examine how race and ethnicity interact with opposition to wind projects in the United States, we aimed to estimate the racial and ethnic composition of opponents. We did this by extracting instances where protesters were named in the article, and feeding these names into a race and ethnicity classification Application Programming Interface. Of the original articles, names were only extracted out of those that were coded as having opposition. The algorithm uses a predetermined set of names and searches for matches. First and last names are extracted during this process. This process produced 9,756 unique names. All extracted names were classified by NamePrism, a well-validated name classifier that has been used in over 200 social scientific research papers (*SI Appendix*).

## Supplementary Material

Appendix 01 (PDF)

## Data Availability

Dataset and replication code data have been deposited in Harvard Dataverse (55).
