# Supplementary material for: Prevalence and predictors of wind energy opposition in North America
Source: Proc Natl Acad Sci U S A. 2023 Sep 25;120(40):e2302313120. doi: 10.1073/pnas.2302313120 (PMC10556604; doi:10.1073/pnas.2302313120)
Supplement: Supplementary file 1 — Appendix 01 (PDF) [file pnas.2302313120.sapp.pdf]

PNAS

# Supporting Information for

## Prevalence and predictors of wind energy opposition in North America

**Leah Stokes, Emma Franzblau, Jessica R. Lovering and Chris Miljanich**

**Corresponding Author name.**
**E-mail: lstokes@ucsb.edu**

**This PDF file includes:**

Tables S1 to S4
SI References

**OLS Regression Tables.** Table S1 shows the full regression results for the four OLS models for USA wind projects: 1) demographics and community ownership; 2) demographics, community ownership and other project characteristics; 3) demographics, project characteristics, region, and partisanship; 4) all variables excluding those missing data (community ownership, turbine height) to avoid dropping units. Midwest is the base category for the geographic dummy variable.

**Table S1. United States of America OLS Regression Models.**

| | OLS | | | |
|---|---|---|---|---|
| | (1) | (2) | (3) | (4) |
| Population Density ($mi^2$, per 1,000) | 0.001 | 0.012 | −0.015 | −0.021 |
| | (0.011) | (0.012) | (0.013) | (0.013) |
| Median Income (per 10,000) | 0.001 | 0.003 | −0.006 | −0.007 |
| | (0.007) | (0.007) | (0.007) | (0.008) |
| Percent White | 0.066 | 0.009 | 0.018 | 0.089 |
| | (0.070) | (0.071) | (0.093) | (0.093) |
| Percent Hispanic/Latino | −0.216*** | −0.306*** | −0.247*** | −0.223** |
| | (0.078) | (0.082) | (0.089) | (0.087) |
| Percent Black | 0.092 | 0.057 | −0.053 | −0.018 |
| | (0.163) | (0.164) | (0.158) | (0.156) |
| Community Ownership (AWEA) | −0.066*** | −0.010 | −0.021 | |
| | (0.023) | (0.027) | (0.026) | |
| Capacity (MW) | | 0.0001 | 0.0001 | 0.001 |
| | | (0.0004) | (0.0004) | (0.0004) |
| Number of Turbines | | 0.001 | 0.001 | 0.0004 |
| | | (0.001) | (0.001) | (0.001) |
| Turbine Height (ft) | | 0.0003** | 0.0003** | |
| | | (0.0002) | (0.0002) | |
| Northeast | | | 0.188*** | 0.188*** |
| | | | (0.042) | (0.043) |
| South | | | −0.016 | −0.018 |
| | | | (0.040) | (0.041) |
| West | | | 0.001 | 0.005 |
| | | | (0.029) | (0.030) |
| Percent GOP Votes | | | −0.107 | −0.098 |
| | | | (0.079) | (0.080) |
| Constant | 0.129* | 0.047 | 0.132 | 0.140* |
| | (0.073) | (0.081) | (0.089) | (0.080) |
| N | 1,155 | 1,154 | 1,154 | 1,176 |
| $R^2$ | 0.025 | 0.040 | 0.073 | 0.082 |
| Adjusted $R^2$ | 0.020 | 0.033 | 0.063 | 0.074 |
| *Note:* | *p<0.1; **p<0.05; ***p<0.01 | | | |

 Leah Stokes, Emma Franzblau, Jessica R. Lovering and Chris Miljanich

Table S2 shows the full regression results for the Canadian wind projects for the three OLS models: 1) demographics only; 2) demographics and project characteristics; 3) demographics, project characteristics, region, and partisanship. Atlantic Canada is the base category for the geographic dummy variable. "Percent Liberal" is the percentage of votes in the 2016 federal election for the Liberal Party. Community ownership of wind projects in Canada was relatively rare and thus was not included in the models.

**Table S2. Canada OLS Regression Models.**

| | *OLS* | | |
|---|---|---|---|
| | (1) | (2) | (3) |
| Population Density ($mi^2$, per 1,000) | 0.026 | −0.095 | −0.036 |
| | (1.294) | (1.712) | (2.084) |
| Median Income (in 10,000) | 0.051** | 0.045** | 0.028 |
| | (0.021) | (0.022) | (0.036) |
| Percent White | 0.006 | 0.001 | 0.007 |
| | (0.007) | (0.009) | (0.008) |
| Capacity (MW) | | 0.001 | 0.001 |
| | | (0.002) | (0.002) |
| Number of Turbines | | 0.0003 | 0.0001 |
| | | (0.004) | (0.004) |
| British Columbia | | | −0.279** |
| | | | (0.138) |
| Canadian Prairies | | | −0.034 |
| | | | (0.182) |
| Ontario | | | 0.262** |
| | | | (0.109) |
| Quebec | | | −0.110 |
| | | | (0.096) |
| Percent Liberal | | | 0.152 |
| | | | (0.255) |
| Constant | −0.783 | −0.295 | −0.904 |
| | (0.757) | (0.967) | (0.966) |
| N | 223 | 216 | 206 |
| $R^2$ | 0.032 | 0.072 | 0.198 |
| Adjusted $R^2$ | 0.018 | 0.050 | 0.157 |
| *Note:* | *p<0.1; **p<0.05; ***p<0.01 | | |

**Additional Details on Hand-Coding of Newspaper Articles.** Each newspaper article was double coded by independent coders. If the original two coders did not agree, the article was adjudicated by a third coder who had not previously read the article. The intercoder reliability between the original coders was 89 percent. The initial pool of positive cases was then reviewed and compared with original newspaper data to ensure that false positives did not exist. False positives were recoded as not being anti-wind. After all articles were coded, they were grouped by plant name, and aggregate measures were constructed for each plant. At the end of this process, a project was coded as experiencing opposition if there were at least three newspaper articles that discussed opposition. We chose this cut off to ensure that we were not over-estimating opposition.

***Associating Articles with Specific Projects.*** For each article, the coders checked whether the plant name assigned to the article was correct. This ensured that the fuzzy matching algorithm converged on the correct wind plant name, and that articles were not incorrectly assigned to the wrong plant. In some cases, the fuzzy matched names were very similar. If so, all plant names that were fuzzy matched to the article were listed as names. If the listed names for an article were very similar, or had a different number listed at the end of the name, the coders coded all listed projects the same. For example, California is home to Alta Wind Energy Center I, and Alta Wind Energy Center II. When coders came across plants with naming conventions such as this, they coded all plants with that name the same, regardless of the plant installation number (i.e, I or II). In cases where the fuzzy matched name did not match the name in the article, or if the article did not mention a plant, coders listed the correct name, or stated that the article did not mention a plant name. Some articles were matched to more than one plant name. When this occurred, the article was duplicated and assigned to each of the names that were fuzzy matched to it. Each duplicated article was then coded according to the single plant name that was associated with it.

***Data on Protest Size.*** We aimed to collect the size of protests if they were estimated in newspaper articles. If an article mentioned the size of a physical protest, we recorded that number. For the American data, 72 articles gave an estimate for protest size, with a median value of 23. For the Canadian data, 31 articles gave an estimate for protest size, with a median value of 34. Our goal was to collect comprehensive data on wind opposition, and pulling protest size estimates from newspaper articles is the only way we could gather an estimate of protest size retroactively. Some studies have found that relative sizes are accurate to an order of magnitude, while others have critiqued estimates for being biased (1–3). Overall, given large communities may turnout more people to a protest, compared to smaller communities, we do not overly rely on this metric in our analysis.

**Operational and Canceled Opposed Projects in the USA.** Here we examine the difference between opposed wind projects that became operational and those that were canceled in the USA. It's important to note these projects were not necessarily canceled because they faced public opposition. Some were likely canceled for economic reasons or constraints on transmission capacity. Notably, of the 197 wind projects we found that faced opposition during this period, only 12% were canceled. We find that opposed projects were more likely to be canceled in areas with higher percentages of White people, and lower percentages of Hispanic people. Larger projects with higher proposed capacity (MW) were also more likely to be canceled—the average size for canceled projects was almost twice as big as those that became operational. Lower population density was also significant in predicting cancellation amongst opposed projects, with canceled projects more likely in more rural areas.

**Table S3. Demographic and Project Characteristics for USA Opposed Wind Projects that were Operational and Canceled**

| | Operational | Canceled | T-statistic |
|---|---|---|---|
| Population Density (per $mi^2$) | 130 | 53 | -1.73* |
| Household Median Income | $50,100 | $51,400 | 0.56 |
| Percent White | 89% | 95% | 4.15*** |
| Percent Hispanic | 6% | 2% | -3.96*** |
| Percent Black | 2% | 1% | -0.80 |
| Percent GOP Votes | 68% | 71% | 1.45 |
| Capacity (MW) | 100 | 194 | 1.82* |
| Number of Turbines | 56 | 89 | 1.40 |
| *N* | 173 | 24 | |

**Race and Ethnicity of Names in Articles in the USA.** We use NamePrism to classify race and ethnicity of opposition for articles in the American data. NamePrism is a classifier that uses name embeddings to predict nationality, ethnicity, and race. The classifier was trained on data that is representative of 90 percent of the World's population. The classifier is shown to outperform similar classifiers in terms of accuracy, and has an F1 score of 0.795 (where a score 1 would be the most accurate). In similar trials, the next best performing classifier had an F1 score of 0.580. This classifier has been used in over 200 social scientific research papers, and we are confident that, while not perfect, it provides an accurate representation of race and ethnicity in cases where we are unable to identify it with primary source data. Ultimately, this is an additional analysis that bolsters our other findings.

As described in the methods section of the paper, we used an API to generate a list of potential names. However, many names included non-human entities, such as wind project names, or other string patterns that resembled human names. To ensure that the final sample only included human names, we processed it against a set of code words. In general, these code words appeared similar to human names, but, in fact, were not. Names that contained any of these code words were eliminated from the sample. An example code word is "Sierra Nevada", which represents the mountainous region of eastern California, but bears resemblance to an individual whose first name is Sierra.

Retained names were fuzzy matched against the original list of wind plant names to ensure that plant names were not inadvertently included in the list of human names. Each human name was checked against the set of plant names. If the human name contained a partial or full string match to the plant name, it was removed from the sample. There were a moderately large number of plant names in the name sample because they were mentioned in close proximity to human names and, as a result, were recognized by the algorithm as human. In total, 2,157 unique human names ended up in the final sample of names.

We elected to post-process the data in this way to ensure that the widest possible set of human names was extracted at the first step, even if it meant non-human names were included as well. While non-human names were classified by the API, they were removed post-classification, and not used in any analysis.

**Table S4. Race and Ethnicity of Opposition in the USA**

| | Probability of Being From a Given Race | | | | |
|---|---|---|---|---|---|
| Race | Hispanic | Asian | Black | American Indian | White |
| *P*(Race) | 1.52 | 1.94 | 3.88 | 0.21 | 92.41 |
| N = 2157 | | | | | |

## References


1. C McPhail, J McCarthy, Who Counts and How: Estimating the Size of Protests. *Contexts* **3**, 12–18 (2004).
2. A Sobolev, M Keith Chen, J Joo, ZC Steinert-Threlkeld, News and Geolocated Social Media Accurately Measure Protest Size Variation. *Am. Polit. Sci. Rev.* **114**, 1343–1351 (2020).
3. E Swank, JD Clapp, Some methodological concerns when estimating the size of organizing activities. *J. Community Pract.* **6**, 49–69 (1999).
